# Supplementary material for: Bacteriophages avoid autoimmunity from cognate immune systems as an intrinsic part of their life cycles
Source: Nat Microbiol. 2024 Apr 2;9(5):1312–24. doi: 10.1038/s41564-024-01661-6 (PMC11087260; doi:10.1038/s41564-024-01661-6)
Supplement: Supplementary file 1 — Reporting Summary [file 41564_2024_1661_MOESM1_ESM.pdf]

nature portfolio

Corresponding author(s): Jakob T. Rostøl & Jose R. Penades

Last updated by author(s): 31/01/2024

# Reporting Summary

Nature Portfolio wishes to improve the reproducibility of the work that we publish. This form provides structure for consistency and transparency in reporting. For further information on Nature Portfolio policies, see our Editorial Policies and the Editorial Policy Checklist.

## Statistics

For all statistical analyses, confirm that the following items are present in the figure legend, table legend, main text, or Methods section.

| n/a | Confirmed | |
|---|---|---|
| ☐ | ☒ | The exact sample size (*n*) for each experimental group/condition, given as a discrete number and unit of measurement |
| ☐ | ☒ | A statement on whether measurements were taken from distinct samples or whether the same sample was measured repeatedly |
| ☐ | ☒ | The statistical test(s) used AND whether they are one- or two-sided<br>*Only common tests should be described solely by name; describe more complex techniques in the Methods section.* |
| ☐ | ☒ | A description of all covariates tested |
| ☐ | ☒ | A description of any assumptions or corrections, such as tests of normality and adjustment for multiple comparisons |
| ☐ | ☒ | A full description of the statistical parameters including central tendency (e.g. means) or other basic estimates (e.g. regression coefficient) AND variation (e.g. standard deviation) or associated estimates of uncertainty (e.g. confidence intervals) |
| ☐ | ☒ | For null hypothesis testing, the test statistic (e.g. *F*, *t*, *r*) with confidence intervals, effect sizes, degrees of freedom and *P* value noted<br>*Give P values as exact values whenever suitable.* |
| ☒ | ☐ | For Bayesian analysis, information on the choice of priors and Markov chain Monte Carlo settings |
| ☒ | ☐ | For hierarchical and complex designs, identification of the appropriate level for tests and full reporting of outcomes |
| ☒ | ☐ | Estimates of effect sizes (e.g. Cohen's *d*, Pearson's *r*), indicating how they were calculated |

*Our web collection on statistics for biologists contains articles on many of the points above.*

## Software and code

Policy information about availability of computer code

| | |
|---|---|
| Data collection | No software was used for data collection |
| Data analysis | No custom code or software was used.<br>Statistical analyses were performed on GraphPad Prism v10.1<br>Clustal Omega v1.2.4 for protein alignments<br>EMBOSS Needle v6.6.0 for calculating protein sequence identity and similarity<br>Alphafold2 was run through Google ColabFold v1.5.2<br>PyMol v2.5.4 for structure visualisation<br>DALI web server (http://ekhidna2.biocenter.helsinki.fi/dali/) (2023) was performed for looking for Tha-1 HEPN domain protein homologues<br>pBLAST 2.14.0 for finding 80α ORF61 homologues/Φ11 ORF54 homologues in phage genomes<br>READemption v0.4.3 RNAseq pipeline for 80α infection RNAseq analysis<br>MAFFT Galaxy v7.508 for protein alignments prior to phylogenetic analysis<br>FASTTREE Galaxy v2.1.10 for phylogenetic analysis<br>Guppy v3.5.1 for base calling during Oxford Nanopore RNA sequencing<br>Geneious Prime 2023 (Biomatters) for read mapping during Oxford RNA sequencing |

For manuscripts utilizing custom algorithms or software that are central to the research but not yet described in published literature, software must be made available to editors and reviewers. We strongly encourage code deposition in a community repository (e.g. GitHub). See the Nature Portfolio guidelines for submitting code & software for further information.

## Data

Policy information about availability of data

All manuscripts must include a data availability statement. This statement should provide the following information, where applicable:

- Accession codes, unique identifiers, or web links for publicly available datasets
- A description of any restrictions on data availability
- For clinical datasets or third party data, please ensure that the statement adheres to our policy

The Oxford Nanopore RNA sequencing data is available through BioProject PRJNA922758. The 80α infection RNAseq data is available from Sequence Read Archive accession code PRJNA1080480. The 80α genome is available through NC_009526, the ΦNM2 genome through DQ530360.1, and the other phage accession codes are listed in Extended Data Excel file 1b. The remaining data is available in the text/figures/extended materials, and raw data and materials/strains upon request from J.T. Rostøl (j.rostoel@imperial.ac.uk) or J.R. Penadés (j.penades@imperial.ac.uk).

## Research involving human participants, their data, or biological material

Policy information about studies with human participants or human data. See also policy information about sex, gender (identity/presentation), and sexual orientation and race, ethnicity and racism.

| | |
|---|---|
| Reporting on sex and gender | N/A |
| Reporting on race, ethnicity, or other socially relevant groupings | N/A |
| Population characteristics | N/A |
| Recruitment | N/A |
| Ethics oversight | N/A |

Note that full information on the approval of the study protocol must also be provided in the manuscript.

# Field-specific reporting

Please select the one below that is the best fit for your research. If you are not sure, read the appropriate sections before making your selection.

☒ Life sciences ☐ Behavioural & social sciences ☐ Ecological, evolutionary & environmental sciences

For a reference copy of the document with all sections, see nature.com/documents/nr-reporting-summary-flat.pdf

# Life sciences study design

All studies must disclose on these points even when the disclosure is negative.

| | |
|---|---|
| Sample size | Pre-determining sample size was not performed for this study. Quantitative experiments were performed three times to allow statistical analysis of significance, which provided robust differences between samples. |
| Data exclusions | We did not exclude any data |
| Replication | All experiments were performed with three biological replicates, unless stated otherwise. All attempts at replication were successful<br>Performed twice - Fig. 4a-c, Extended Data Fig. 2d<br>Performed once - Fig. 3c, d, Extended Data. Fig. 1e, g, Extended Data. Fig. 4a-b, and RNAseq experiments |
| Randomization | Samples were not randomised since we employed well-defined experimental strains with isogenic genetic backgrounds, comparing effects of single, known variables. No animals or patients were used in this study. |
| Blinding | Blinding was not performed since we used well-defined experimental strains, and results had strong effect sizes. The same researcher prepared samples and performed experiments. No animals or patients were used in this study. |

# Reporting for specific materials, systems and methods

We require information from authors about some types of materials, experimental systems and methods used in many studies. Here, indicate whether each material, system or method listed is relevant to your study. If you are not sure if a list item applies to your research, read the appropriate section before selecting a response.

## Materials & experimental systems

| n/a | Involved in the study |
|---|---|
| ☐ | ☒ Antibodies |
| ☒ | ☐ Eukaryotic cell lines |
| ☒ | ☐ Palaeontology and archaeology |
| ☒ | ☐ Animals and other organisms |
| ☒ | ☐ Clinical data |
| ☒ | ☐ Dual use research of concern |
| ☒ | ☐ Plants |

## Methods

| n/a | Involved in the study |
|---|---|
| ☒ | ☐ ChIP-seq |
| ☒ | ☐ Flow cytometry |
| ☒ | ☐ MRI-based neuroimaging |

# Antibodies

| | |
|---|---|
| Antibodies used | Monoclonal anti-FLAG M2-Perixodase (HRP) antibody from mouse, from Sigma-Aldrich. Catalogue number A8592, lot number SLCF0816 (1/1,000 dilution)<br>Custom anti-GlmM polyclonal antibodies from rabbit, from Covalabs (project number 1846011) (1/2,500 dilution)<br>Anti-rabbit IgG, HRP-linked antibody, from Cell Signal. Catalogue number 7074S, lot number 26 (1/10,000 dilution) |
| Validation | The anti-GlmM antibody was validated by Western blot in prof. Angelika Gründling's laboratory (Imperial College London), where native S. aureus GlmM and His-tagged S. aureus GlmM produced a clean signal at the right size, and E. coli GlmM expressed in S. aureus produced no signal. |
