# Supplementary figures and images for: Bacteriophages avoid autoimmunity from cognate immune systems as an intrinsic part of their life cycles
Source: Nat Microbiol. 2024 Apr 2;9(5):1312–24. doi: 10.1038/s41564-024-01661-6 (PMC11087260; doi:10.1038/s41564-024-01661-6)

Fig 3c Agilent bioanalyzer

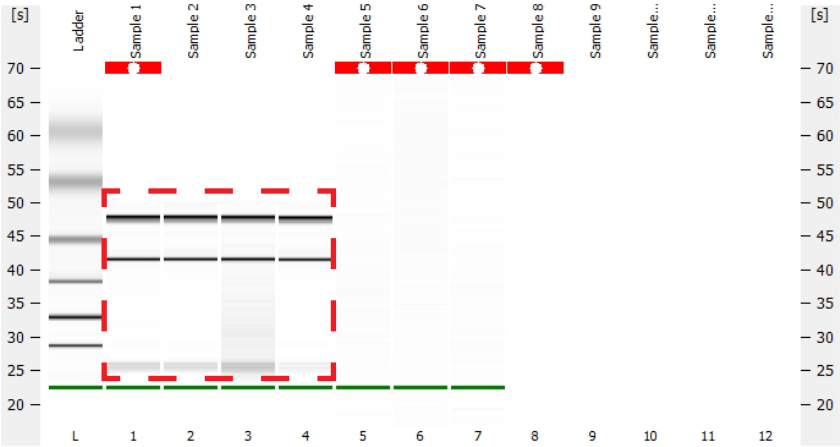

Fig 3g Southern blot

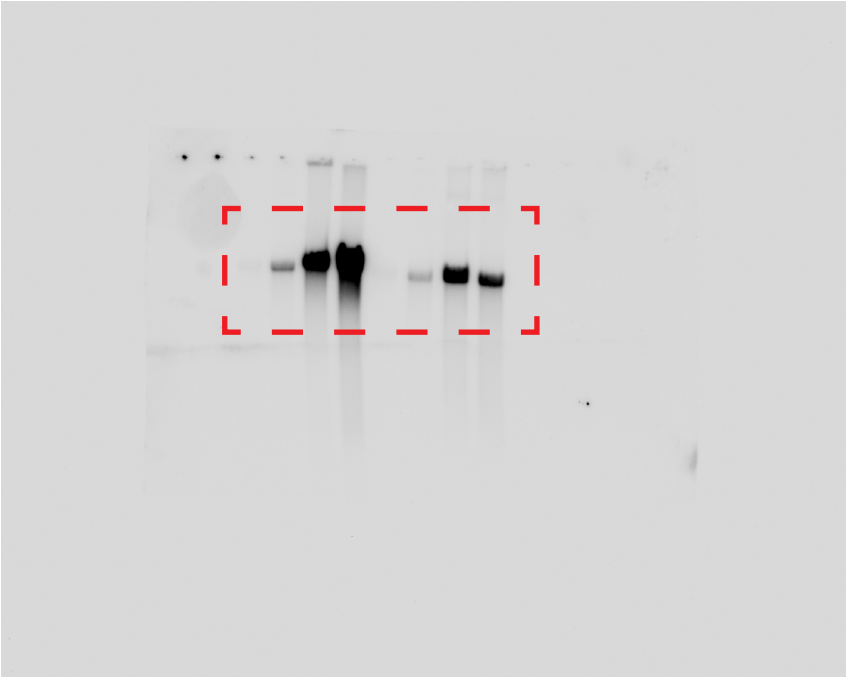

Supplement: Supplementary file 6 — Agilent Bioanalyzer showing RNA degradation, and southern blot raw image. [file 41564_2024_1661_MOESM6_ESM.pdf]

Extended Data Fig. 1e, g, non-quantitative PCR

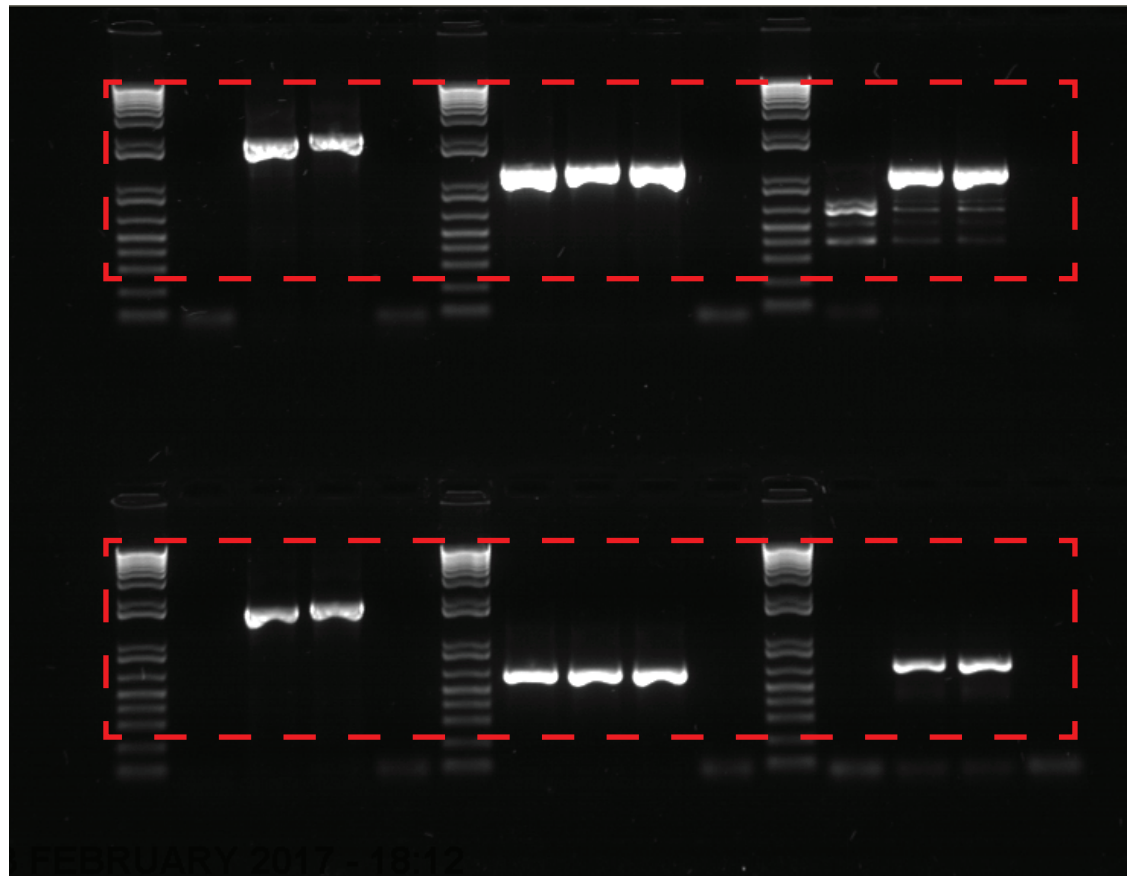

Supplement: Supplementary file 10 — Non-quantitative PCR agarose gel raw image. [file 41564_2024_1661_MOESM10_ESM.pdf]

Extended Data Fig. 4b, Western blot

Tha-1/Tha-1<sup>H270A</sup>

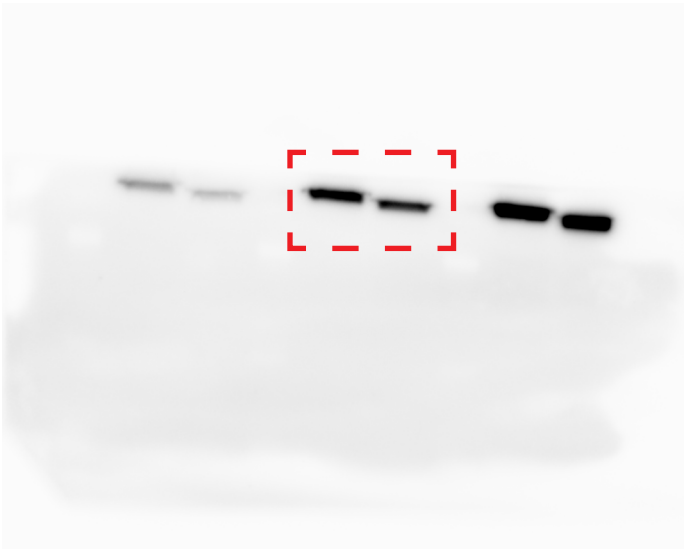

GlmM

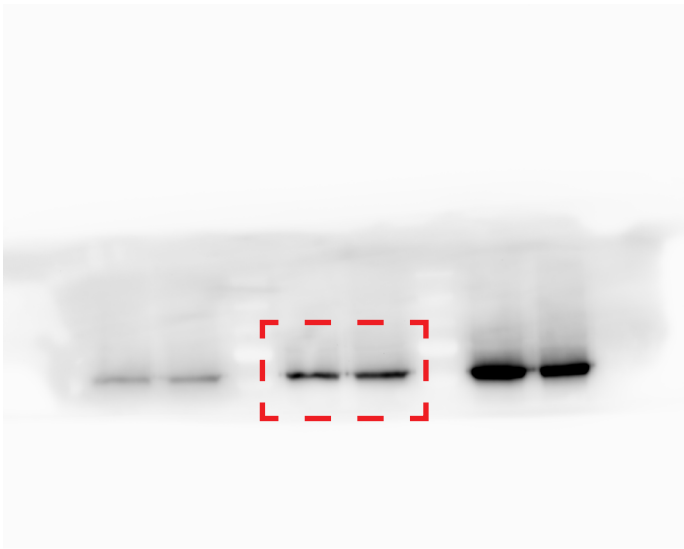

Supplement: Supplementary file 13 — Raw images for western blot. [file 41564_2024_1661_MOESM13_ESM.pdf]
